# The Bergman vs. “Dr.” Volker Case Settled

**Published:** 1877-04

**Authors:** 


### The Bergman vs. "Dr." Volker Case Settled.

" The Mills of the Gods grind slow, but they grind exceedingly fine."

Bergman so well known to the profession in Buffalo, and to all attentive readers of the October and November numbers of our JOURNAL, is dead. His various law suits terminating unsuccessfully, life at last became too burdensome, and he took the matter into his own hands. He managed, we are told, by aid of the hook and rope prepared to reduce his dislocated hip, (?) to hang himself until dead. He gave his body to the doctor, his soul to God, and ended his life March 2d, 1877, upon his own improvised gallows, and the doctors all said amen. The *post mortem*, made in presence of nearly the entire Medical Profession of Buffalo, and the attorneys in the recent trial for malpractice, showed *fracture of the neck of femur*, the head of bone resting in the acetabulum with ligamentous union and absorption of neck, and made the medical testimony reported in full in our October, and the correspondence published in our November number upon Bergman vs. "Dr." Volker of the greatest possible interest and significance, now worthy careful re-perusal. Not as showing anything new or uncommon in the symptoms or nature of hip-joint injuries, but as showing how careful we should be in the selection of medical advisors, how attentively we should scrutinize our own conclusions and how we should remember that *experience* teaches many valuable lessons. In all respects the trial and testimony in the case of Bergman vs. Volker, is one of the most suggestive and remarkable of any Medico-legal suit in our court record. Reading the testimony in the light of the *post mortem* examination is painfully suggestive, showing how it is possible, for even an honest man, to make his facts correspond with, and sustain his theories, never himself finding out that his theories were made first and his facts, imaginary and unreal.

